# Acute kidney injury due to ciprofloxacin for treatment of acute pyelonephritis

**Published:** 2015-10-09

**Authors:** Beuy Joob, iroj Wiwanitkit

**Affiliations:** ^1^Sanitation 1 Medical Academic Center, Bangkok, Thailand; ^2^Hainan Medical University, Haikou, China; ^3^Faculty of Medicine, University of Nis, Serbia

**Keywords:** Ciprofloxacin, Renal failure, Acute kidney injury

**Implication for health policy/practice/research/medical education:**

Ciprofloxacin is an important widely used antibiotic. Similar to any antibiotic, the adverse effect can be expected. Here, the authors correspond to previous case report on this topic. A similar case is hereby shown. The discussion on epidemiology and prevention of this problem is also available in this article.

## Introduction

Ciprofloxacin is an important widely used antibiotic. It is classified into the fluoroquinolone group and widely used for treatment of infection of gastrointestinal tract, respiratory tract and genitourinary tract (1). Similar to any antibiotic, the adverse effect can be expected. Ball noted that “the most common adverse effects involve the gastro-intestinal system (2%-8% of patients treated) and usually comprise nausea, vomiting, diarrhea and abdominal discomfort (2).” The renal side effects of ciprofloxacin is an interesting topic in nephropharmacology. Here, the authors correspond to previous case report on this topic. A similar case is hereby shown. The discussion on epidemiology and prevention of this problem is also available in this article.

## Case report on “crystal-induced acute kidney injury due to ciprofloxacin”

The recent case report published by Khan et al on “crystal-induced acute kidney injury due to ciprofloxacin” is very interesting (3). Khan et al reported a case of “an elderly woman who developed oliguric acute kidney injury (AKI) after receiving oral and intravenous ciprofloxacin in a 48-hour period (3).” Here, the authors would like to share an experience on a similar case. The case is also an elderly female patient. She is a known diabetic patient firstly presented with the problem of acute pyelonephritis. The case was treated by oral ciprofloxacin treatment. While the clinical symptoms (fever, pain, dysuria), decreased and disappeared, the patient developed more turbid urine within 1 week. Focusing on urinalysis, from first visiting, the white blood cells (WBC) in urine shift from 100/HPF to more than 300/HPF and bacteria from 4 + to no bacteria. The renal function test of this case showed BUN 19 mg/dl and serum creatinine 0.9 mg/dl. The estimated glomerular filtration rate (eGFR) = 36 cc/min (1 month before result, BUN 16 mg/dl, creatinine 0.6 mg/dl, eGFR = 68 cc/min). The case was suspected for ciprofloxacin induced renal problem. Any drugs were stopped and the conservative treatment is done. One week follow-up, the patient had no problem; fever, pain, dysuria, and her urine was clear without WBC and bacteria. At this time, the laboratory results showed BUN 17 mg/dl and creatinine 0.6 mg/dl and eGFR=64 cc/min. Khan et al mentioned that “conservative measures including intravenous hydration and avoidance of alkalinization of the urine can reverse this condition if applied in time.” In their case, the early diagnosis was done and dramatically improvement could be observed with conservative therapy. While ciprofloxacin can be useful for management of urinary tract infection in the elderly, its unwanted effect should be aware and early managed (4,5).

## Crystal-induced acute kidney injury: epidemiology, management and prevention

Stahlmann and Lode noted that “chronic kidney diseases, concomitant administration of corticosteroids and age >60 years are known risk factors” for adverse effect of ciprofloxacin administration, especially for the elderly (6). In the presented case, the patient is also an elderly female. Focusing on the specific problem, crystal-induced AKI, Stratta et al said that “the probability that ciprofloxacin crystal nephropathy would happen in humans was believed to be very low on the basis of previous data presentation that ciprofloxacin crystalluria depended on a urine pH greater than 6.8 (7).” The exact incidence of crystal-induced AKI has never been systematically evaluated but it should be very rare since there are very few case reports on this problem (3,7-9). Focusing on the reported cases (3,7-9), most patients are elderly female. The problem can be seen in few days after getting ciprofloxacin. The renal biopsy is usually the effective diagnostic tool but invasive. However, in some case the diagnosis can be made by observation of “an amelioration in kidney function that followed the discontinuation of ciprofloxacin (10),” such as our discussed case.

Focusing on renal pathology, acute interstitial nephritis with the depositing of a brown-yellowish ciprofloxacin salt is the main finding (8). For the cases, the standard management of acute renal injury can be applied. As noted by Khan et al (3), “conservative measures comprising intravenous hydration and avoidance of alkalinization of the urine” is the key words for management of the cases. It is no doubt that prevention of the problem is the appropriate hydration during the use of ciprofloxacin. Also, since the problem can be relative to over dosage, the meticulous prescription is needed for any elderly patients (11).

## Conclusion

Ciprofloxacin is an important widely used antibiotic. Similar to any antibiotic, the adverse effect can be expected. Here, the authors correspond to previous case report on this topic. A similar case is hereby shown. The discussion on epidemiology and prevention of this problem is also available in this article.

## Authors’ contribution

All authors wrote the paper equally.

## Conflicts of interest

The authors declared no competing interests.

## Ethical considerations

Ethical issues (including plagiarism, data fabrication, double publication) have been completely observed by the authors.

## Funding/Support

None.
